# Type 5 adenylyl cyclase disruption leads to enhanced exercise performance

**DOI:** 10.1111/acel.12401

**Published:** 2015-10-01

**Authors:** Dorothy E. Vatner, Lin Yan, Lo Lai, Chujun Yuan, Laurent Mouchiroud, Ronald E. Pachon, Jie Zhang, Jean‐Guillaume Dillinger, Riekelt H. Houtkooper, Johan Auwerx, Stephen F. Vatner

**Affiliations:** ^1^ Department of Cell Biology & Molecular MedicineNew Jersey Medical SchoolRutgers UniversityNewarkNJUSA; ^2^ Laboratory of Integrative and Systems PhysiologyEcole Polytechnique Fédérale de Lausanne (EPFL)LausanneSwitzerland

**Keywords:** antioxidant defense, exercise, mitochondrial biogenesis, skeletal muscle, type 5 adenylyl cyclase

## Abstract

The most important physiological mechanism mediating enhanced exercise performance is increased sympathetic, beta adrenergic receptor (β‐AR), and adenylyl cyclase (AC) activity. This is the first report of decreased AC activity mediating increased exercise performance. We demonstrated that AC5 disruption, that is, knock out (KO) mice, a longevity model, increases exercise performance. Importantly for its relation to longevity, exercise was also improved in old AC5 KO. The mechanism resided in skeletal muscle rather than in the heart, as confirmed by cardiac‐ and skeletal muscle‐specific AC5 KO's, where exercise performance was no longer improved by the cardiac‐specific AC5 KO, but was by the skeletal muscle‐specific AC5 KO, and there was no difference in cardiac output during exercise in AC5 KO vs. WT. Mitochondrial biogenesis was a major mechanism mediating the enhanced exercise. SIRT1, FoxO3a, MEK, and the anti‐oxidant, MnSOD were upregulated in AC5 KO mice. The improved exercise in the AC5 KO was blocked with either a SIRT1 inhibitor, MEK inhibitor, or by mating the AC5 KO with MnSOD hetero KO mice, confirming the role of SIRT1, MEK, and oxidative stress mechanisms. The *Caenorhabditis elegans* worm AC5 ortholog, *acy‐3* by RNAi, also improved fitness, mitochondrial function, antioxidant defense, and lifespan, attesting to the evolutionary conservation of this pathway. Thus, decreasing sympathetic signaling through loss of AC5 is not only a mechanism to improve exercise performance, but is also a mechanism to improve healthful aging, as exercise also protects against diabetes, obesity, and cardiovascular disease, which all limit healthful aging.

## Introduction

Exercise is central to longevity and more importantly healthful aging, as it is well recognized to protect against diseases that limit longevity and compromise healthy lifespan, most importantly obesity, diabetes, and cardiovascular disease, but also hypertension, cancer, osteoporosis, depression, and dementia (Lee & Paffenbarger, 2000; Gremeaux *et al*., 2012; Reimers *et al*., 2012). Conversely, diminished exercise capacity is one of the first signs of all cardiovascular diseases, as well as other diseases, and improved exercise capacity is one of the first signs that therapy is effective. The most widely recognized physiological mechanism to improve exercise performance is enhanced sympathetic and beta adrenergic receptor stimulation and increased adenylyl cyclase (AC) activity (Esposito *et al*., 2008), whereas reducing sympathetic stimulation is generally thought to impair exercise performance. We found that inhibiting beta adrenergic signaling at the level of AC, by disrupting the AC isoform type 5 (AC5), that is, AC5 knock out (KO), enhances longevity (Yan *et al*., 2007). In view of the above, it becomes a natural question as to whether inhibiting AC5 improves exercise performance. Accordingly, the central goal of this investigation was to examine exercise performance in the AC5 KO. An important adjunct was to examine this in old AC5 KO mice. Interestingly, most mechanisms of longevity are only studied in young mice and assumed to persist as the animals reach old age.

It was then important to understand the molecular signaling linking the AC5 loss of function to improved exercise. As enhanced exercise can result from mechanisms originating in either the heart or skeletal muscle, we examined cardiac and muscle function and also utilized tissue‐specific KOs in the heart and skeletal muscle. Skeletal muscle mitochondrial function (Yamamoto *et al*., 2011), mitochondrial biogenesis (Irrcher *et al*., 2003), and resistance to oxidative stress (Nishiyama *et al*., 1998; Fisher‐Wellman *et al*., 2009; Ryan *et al*., 2010) were also examined, as these mechanisms mediate exercise performance (Rockl *et al*., 2007). The SIRT1, FOXO3a, and MEK pathways, key to mitochondrial biogenesis and protection against oxidative stress, were also examined (Wu *et al*., 1999; Lagouge *et al*., 2006; Gurd *et al*., 2009; Menzies *et al*., 2013; Li *et al*., 2015; Smith *et al*., 2015). As one of the most potent mechanisms limiting longevity is increased oxidative stress (Gemma *et al*., 2007), MnSOD levels were examined and exercise was examined in AC5 KO mice mated with MnSOD heterozygous (*MnSOD* ^*+/−*^) mice. The evolutionary conservation of the AC5/MnSOD pathway was demonstrated in the worm, *Caenorhabditis elegans* AC5 ortholog*,* which, like the AC5 KO mouse, linked fitness and lifespan extension, testifying further to the central role of AC5 inhibition in regulation of mitochondrial and muscle function resulting in enhanced exercise.

## Results

### Enhanced exercise capacity in AC5 KO mice is not due to improved cardiac function but rather due to improved skeletal muscle function

**Figure 1:**
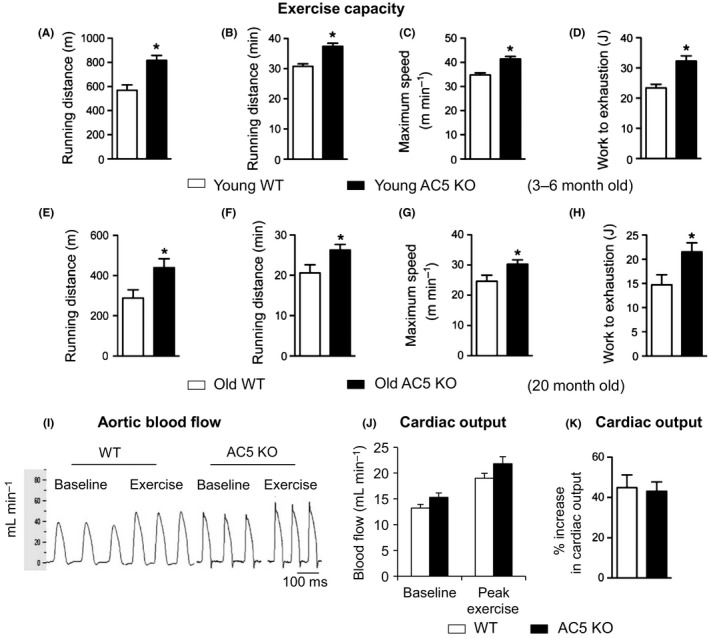
Exercise capacity is enhanced in young and old AC5 systemic KO mice and enhanced exercise capacity in AC5 KO mice is not due to improved cardiac function but rather due to improved muscle function. Exercise capacity is enhanced in young (A–D) (*n* = 11; 3–6 months old) and old (E–H) (*n* = 8; 20 months old) AC5 KO mice, as reflected by running longer distances, over shorter time, achieving higher speed, and doing more work. *P <* *0.01 vs. WT. Although body weights tended to be less in AC5 KO, there was no relationship between body weight and exercise capacity in this study between the groups, with further support by the differences in work, which takes into account body weight. (I) A representative recording is shown for stroke volume and cardiac output in chronically instrumented, conscious mice, using an implanted transonic flow probe on the ascending aorta. AC5 KO and WT increased stroke volume and cardiac output similarly. (J) Blood flow values, shown in mL min^−1^, were similar in AC5 WT and AC5 KO both at baseline and peak exercise. (K) % increase in cardiac output in response to exercise was similar in WT and AC5 KO mice (*n* = 7–8). AC, adenylyl cyclase; KO, knock out.

AC5 systemic KO mice ran significantly, *P *<* *0.01, longer than WT littermates in both distance and time and also 17% faster, resulting in 38% greater work to exhaustion (Fig. 1A–D). Exercise capacity was also significantly improved, *P* < 0.01, in older AC5 KO mice compared to WT (20 months) (Fig. 1E–H).

To investigate whether the enhanced exercise capacity in AC5 KO mice was due to better cardiac function, we measured cardiac output in chronically instrumented, conscious mice, at baseline and during exercise. There were no significant differences between AC5 KO and WT in ether baseline or peak exercise in heart rate (baseline: 646 ± 23 vs. 700 ± 13 beats min^−1^; peak exercise: 764 ± 18 vs. 729 ± 20 beats min^−1^), stroke volume (baseline: 21.8 ± 1.1 vs. 20.7 ± 1.4 μl; peak exercise: 28.5 ± 1.4 vs. 26.2 ± 1.4 μL), and cardiac output (baseline:15.3 ± 1.0 vs. 13.2 ± 0.7 mL min^−1^; peak exercise: 21.8 ± 1.4 vs. 19.0 ± 0.8 mL min^−1^) (Fig. 1I–K), indicating that the mechanism for improvement in exercise did not reside at the level of the heart, but more likely in skeletal muscle performance. Furthermore, we measured VO2 during exercise, which was significantly increased at peak exercise in AC5 KO vs. WT (143 ± 5.5 vs. 130 ± 4.4 mL kg^−1^ min^−1^, *P *<* *0.05), suggesting that AC5 KO mice take in more oxygen and deliver it to the muscles for better exercise performance.

**Figure 2:**
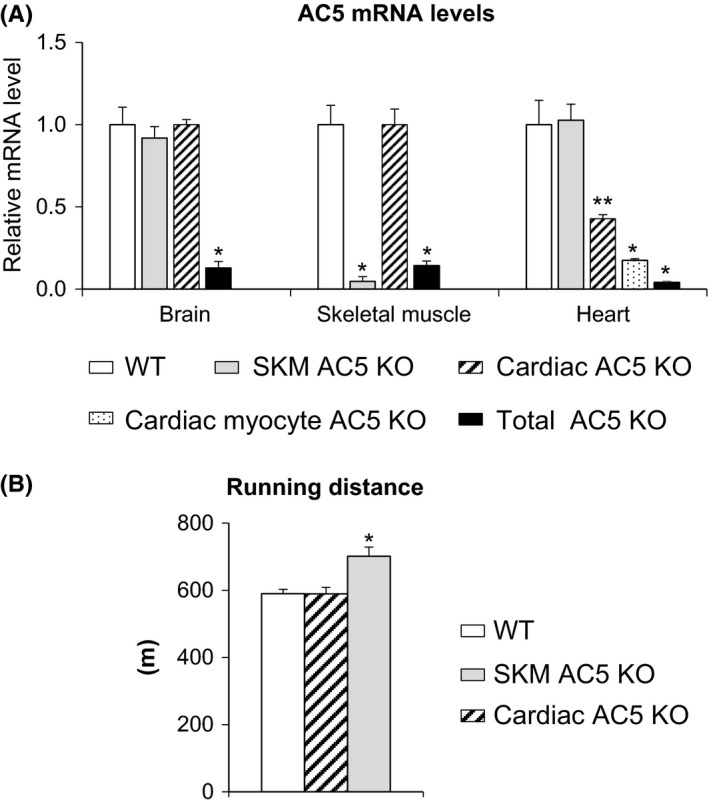
Cardiac and Skeletal Muscle‐specific Knockouts. (A) Relative mRNA expression of AC5 gene in the brain, skeletal muscle, and heart from WT, AC5 in the total AC5 KO was reduced in all tissues studied, but not in WT. The skeletal muscle and cardiac‐specific KO's also showed no reduction in the tissues where the AC5 was not disrupted, but there was specific reduction in the tissues where the AC5 was disrupted. In the cardiac‐specific AC5 KO, the levels were reduced in the heart, but not as completely as in the total body knockout. One explanation is that over 70% of cells in the heart are not myocytes. Accordingly, when the myocytes alone were examined, there was more reduction in AC5. *P <* *0.01 vs. WT and **P <* *0.05 vs. WT using one‐way ANOVA;* n* = 6/group. Results are expressed as the mean ± SEM. (B) Running distance was similar in cardiac‐specific AC5 KO (*n* = 7), compared to WT, but was significantly greater, *P < 0.01, in skeletal muscle AC5 KO (*n* = 6) compared to WT and cardiac‐specific AC5 KO. WT was combined from both groups, as they were similar (*n* = 11). AC, adenylyl cyclase; KO, knock out.

To further confirm these results, we created both cardiac‐specific and skeletal muscle‐specific (SKM) AC5 KO mice. The RNA levels were reduced similar to that of total body AC5 KO in the tissue where the AC5 was specifically disrupted (Fig. 2); however, the cardiac‐specific KO did not show complete loss of AC5, most likely due to the fact that more than 70% of cells in the heart are not myocytes. Examining only the cardiac myocytes in the cardiac‐specific AC5 KO showed more complete elimination of AC5 (Fig. 2A). In contrast to the total body AC5 KO, the cardiac‐specific AC5 KO (*AC5* ^*cardiac−/−*^) did not run faster or longer than WT (Fig. 2B), but the running distance of SKM AC5 KO (*AC5* ^*skm−/−*^) mice was 19% greater, *P < 0.01 vs. WT mice, indicating that the lack of AC5 in skeletal muscle mediates the enhanced exercise capacity (Fig. 2B).

### Increased mitochondrial content in skeletal muscle of AC5 KO mice

**Figure 3:**
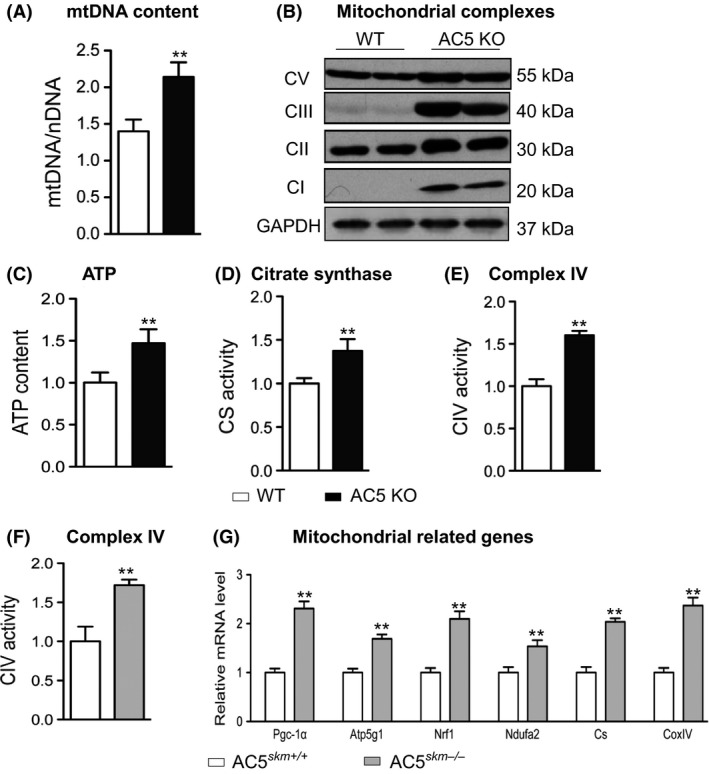
Increased mitochondrial content and activity in skeletal muscle of AC5 KO mice. (A) Increased mtDNA/nuclDNA ratio in AC5 KO gastrocnemius; *n* = 5. (B) Western blotting showing the measurement of the four complexes of the mitochondrial electron transport chain in quadriceps muscles of mice of the indicated genotype; *n* = 4. (C) Skeletal muscle of AC5 KO mice contains more ATP;* n* = 9. (D) Higher citrate synthase activity was present in skeletal muscle of AC5 KO mice; *n* = 12. *E and F*, higher complex IV activity was present in the gastrocnemius muscle of systemic AC5 KO (E) and AC5 SKM KO (F) mice; *n* = 6–8. (G), Relative mRNA expression of mitochondria‐related genes in the gastrocnemius muscle of AC5 SKM KO is shown; *n* = 6. **P <* *0.05 vs. WT using Student's t‐test. Results are expressed as the mean ± SEM. AC, adenylyl cyclase; KO, knock out.

The ratio between mitochondrial versus nuclear DNA (Fig. 3A) and mitochondrial protein content (Fig. 3B) was significantly increased in the gastrocnemius muscle of AC5 KO mice. ATP content, citrate synthase activity, and complex IV activity were increased by ~50% in skeletal muscle of AC5 KO (Fig. 3C–E), a finding confirmed in AC5 SKM KO mice (Fig. 3F). Consistent with enhanced mitochondrial number, mRNA levels of mitochondrial genes (*Atp5 g1, Nrf‐1, Ndufa2, citrate synthase,* and *Cox IV*) were increased in the skeletal muscle of AC5 SKM KO mice (Fig. 3G). These results indicate that mitochondrial biogenesis was the underlying mechanism in the enhanced exercise capacity of AC5 KO mice.

### Knockdown of AC5 in skeletal muscle myoblasts induces increased mitochondrial content

**Figure 4:**
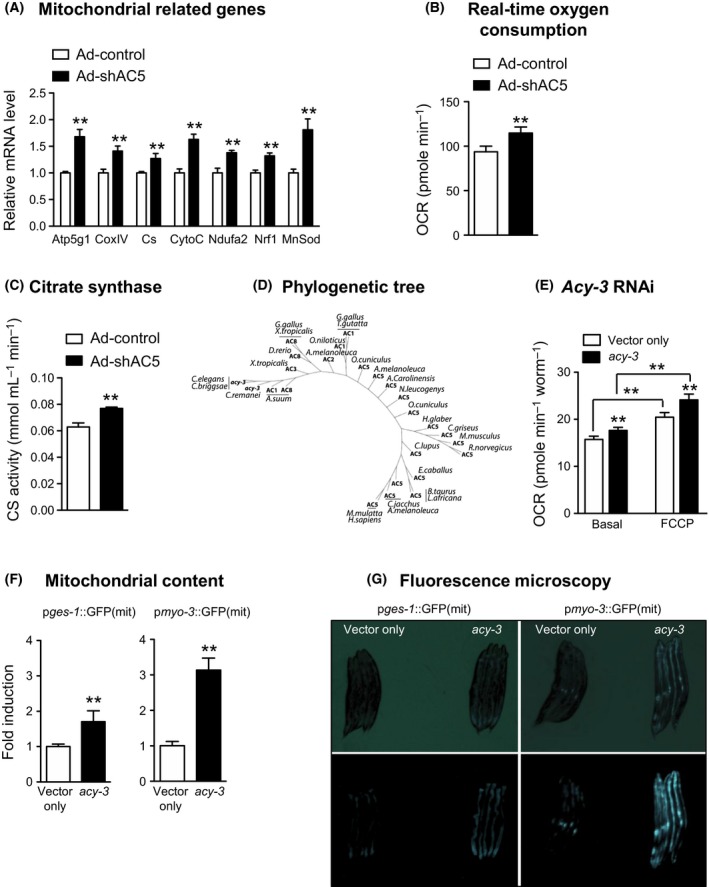
Increased mitochondrial activity in AC5 KD myoblast cells and *Caenorhabditis elegans*. (A) Relative mRNA expression of mitochondria‐related genes in L6 AC5 KD cells; *n* = 3. (B) Real‐time oxygen consumption measured by Seahorse extracellular flux analyzer. AC5 KD cells consumed more oxygen compared with control cells; *n* = 9. (C) Citrate synthase activity was increased in AC5 KD cells; *n* = 3. **P <* *0.05 vs. control using Student's t‐test. Results are expressed as the mean ± SEM. (D) Phylogenetic tree was made by blasting the protein sequence of *acy‐3* against several species. (E) Mitochondria in *C. elegans* increases basal and uncoupled respiration (Seahorse respirometry). FCCP was injected at 10 mm. (F‐G) Both intestinal (pges‐1::GFP(mit)) and muscle (pmyo‐3::GFP(mit)) mitochondrial content are increased after *acy‐3* RNAi as revealed by fluorescence quantification (F) and microscopy imaging (G). Results are expressed as the mean ± SEM. **P <* *0.05 vs. control using Student's t‐test (A, C, D, G) and one‐way ANOVA (F). AC, adenylyl cyclase; GFP, green fluorescent protein.

To confirm that the effects on muscle physiology that were observed in the germline and SKM AC5 KO mice were cell autonomous, we investigated the effects of knockdown (KD) AC5 in myoblast cells *in vitro*. The mRNA levels of several mitochondrial proteins were all significantly increased in AC5 KD myoblasts (Fig. 4A). The mRNA levels of nuclear respiratory factor 1 (*Nrf‐1*), an important mitochondria DNA transcription regulator, and manganese superoxide dismutase 2 (*MnSOD*), a mitochondrial enzyme involved in antioxidant defense, were also increased in AC5 KD myoblasts. In line with these gene expression changes, the oxygen consumption rate, which reflects the mitochondrial metabolic rate, was increased by ~20% in the AC5 KD myoblast cells (Fig. 4B). Likewise, citrate synthase activity was also ~20% higher in the AC5 KD cells (Fig. 4C). In combination, these data support the idea that disruption of AC5 in skeletal muscle myoblasts induced mitochondrial biogenesis in a cell autonomous manner.

### Deletion of AC5 increased mitochondrial function in *Caenorhabditis elegans*

Phylogenetic studies indicated that the AC protein family is conserved throughout evolution. In worms, the AC protein family consists of four members named *acy‐1*,* acy‐2*,* acy‐3,* and *acy‐4*. According to the *C. elegans* worm database (www.wormbase.org), *acy‐1* encodes an adenylyl cyclase that is most closely related to the mammalian isoform type 9, *acy‐2* to the isoform type 2 and 4, *acy‐3* to the isoform type 5, and *acy‐4* to the isoform 5 and 6. *acy‐4* has been described to be required for meiotic maturation (Govindan *et al*., 2009), while *acy‐3* seems to be involved in metabolic function as inactivation of this gene by RNAi robustly reduces fat content in wild‐type worms (Ashrafi *et al*., 2003). We confirmed by BLAST (Basic Local Alignment Search Tool) analysis that *acy‐3* is a close worm homolog of the mouse AC5 gene, with 44% homology in its amino acid sequence (Fig. 4D). Oxygen consumption rate increased in worms fed with *acy‐3* RNAi, compared with worms fed with empty vector—an effect that was evident both in basal and uncoupled conditions (Fig. 4E). Transgenic worms expressing green fluorescent protein (GFP) in the mitochondria (Benedetti *et al*., 2006) demonstrated a 1.5‐fold and threefold increase in GFP signal, respectively, in intestinal and muscle mitochondria in *acy‐3* RNAi (Fig. 4F,G), indicating a conserved function of inhibiting AC5 throughout evolution, as the AC5 homolog in *C. elegans* also improves mitochondrial metabolism.

### Reduced oxidative stress also contributed to AC5 KO‐enhanced exercise

**Figure 5:**
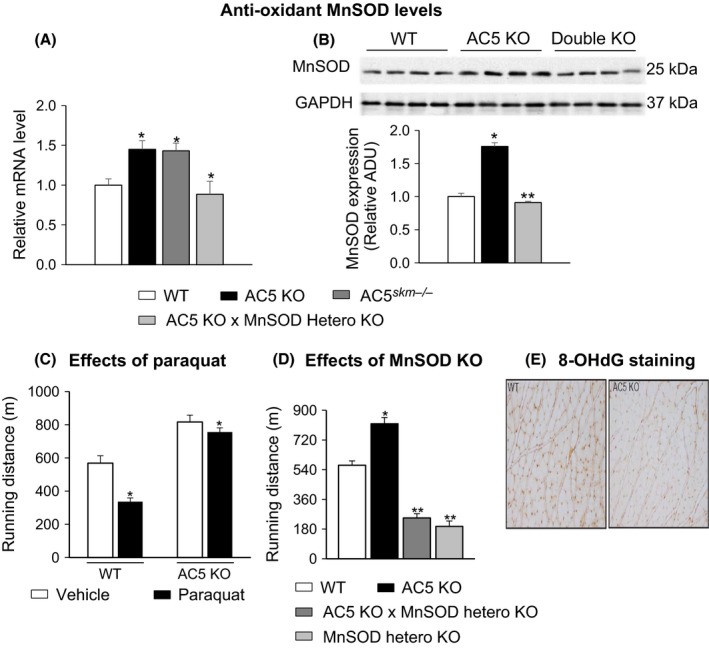
Protection from oxidative stress in AC5 KO mice contributed to the enhanced exercise capacity. (A) Relative MnSOD mRNA levels were increased in the skeletal muscle in systemic AC5 KO and AC5 SKM KO; mating AC5 KO mice with heterozygous MnSOD KO abolished increased MnSOD mRNA levels in AC5 KO;* n* = 6. (B) MnSOD expression was increased in the skeletal muscle from AC5 KO compared with WT mice; mating AC5 KO mice with heterozygous MnSOD KO abolished increased MnSOD in AC5 KO *n* = 4. (C) Paraquat reduced exercise capacity significantly in WT, but not in AC5 KO. *P <* *0.01 vs. WT treated with vehicle using one‐way ANOVA *; n* = 6. (D) Mating AC5 KO mice with heterozygous MnSOD KO abolished enhanced exercise capacity in AC5 KO mice. (E) 8‐OHdG staining of gastrocnemius muscle sections after paraquat treatment showed less staining in AC5 KO. *P <* *0.01 vs. WT and **P <* *0.05 vs. AC5 KO using one‐way ANOVA;* n* = 5. Results are expressed as the mean ± SEM. AC, adenylyl cyclase; KO, knock out.

Protection against oxidative stress is often linked with improved exercise tolerance (Nishiyama *et al*., 1998; Fisher‐Wellman *et al*., 2009; Ryan *et al*., 2010). Paraquat, which increases oxidative stress, reduced WT exercise capacity by 40%, and significantly less, *P < 0.05*, that is, by only 7% in AC5 KO (Fig. 5C) and induced ~40% less oxidative DNA damage in AC5 KO muscles (Fig. 5E). We further examined the antioxidant, MnSOD, in the gastrocnemius muscle and observed increased mRNA and protein levels of MnSOD, which protects against oxidative stress (Fig. 5A,B). To block this mechanism, we mated AC5 KO mice with *MnSOD* heterozygous (*MnSOD* ^*+/−*^) mice. The bigenic mice had significantly reduced MnSOD levels (Fig 5B) and reduced exercise capacity compared to AC5 KO mice (Fig. 5D). Furthermore, 8‐OHdG staining, an indicator of oxidative stress, was reduced in AC5 KO (Fig. 5E). These results indicate clearly that reduced oxidative stress is involved in the enhanced exercise performance of AC5 KO.

### Studies of oxidative stress in the worms supported the findings in the AC5 KO mouse. Enhanced movement and antioxidative defense in *acy‐3* ^*−/−*^ worms is mediated by SOD‐3

**Figure 6:**
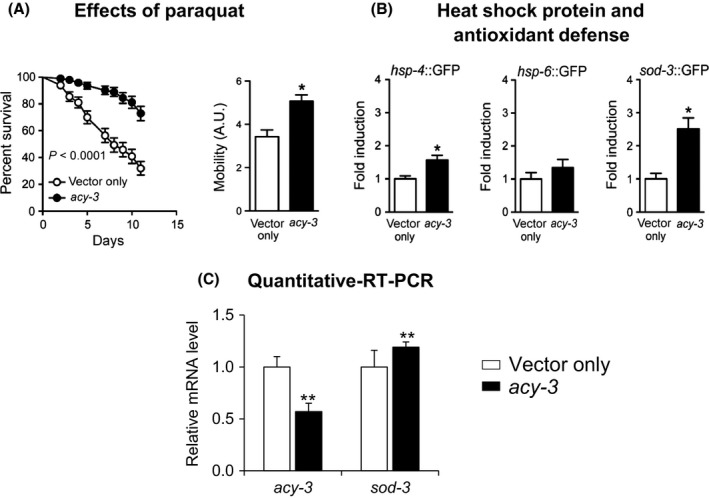
Increased *sod‐3* underlies the fitness and antioxidant defense of *acy‐3* RNAi worms. (A) Resistance to oxidative stress and mobility upon paraquat treatment is increased after *acy‐3* RNAi compared to worms treated with vehicle only. (B) Heat‐shock protein (*hsp‐4*) and antioxidant defense (*sod‐3*), but not markers of the mitochondrial unfolded protein response, are increased upon *acy‐3* RNAi as revealed by fluorescence quantification. *P <* *0.01 using Student's *t*‐test. (C) Quantitative RT‐PCR analysis of *acy‐3* RNAi treated worms confirms *acy‐3* knockdown and shows increased expression of *sod‐3* in mitochondrial oxidative metabolism. **P <* *0.05 Student's t‐test. Results are expressed as the mean ± SEM.

We then analyzed whether *acy‐3* in worms had a similar functional impact on fitness. *acy‐3 RNAi* ^*−/−*^ worms exposed to paraquat from the L4 larval stage exhibited more than 70% survival and greater mobility after 10 days compared to <35% survival for the control worms fed with empty vector (Fig. 6A). We examined the expression of several GFP‐reporter worms that are specific for certain stress pathways, including *hsp‐4* which is induced during endoplasmic reticulum stress, *hsp‐6* which is specifically expressed in response to mitochondrial stress (Yoneda *et al*., 2004), and *sod‐3* which is the homolog of the mitochondrial superoxide dismutase SOD2 involved in detoxification of reactive oxygen species (ROS) (Honda & Honda, 1999). In line with our data in AC5 KO mice, these GFP fluorescence‐based assays indicated that the increased resistance to oxidative stress is mainly a consequence of *sod‐3* overexpression, an observation which was also supported by qPCR experiments showing an induction in *sod‐3* expression (Fig. 6B,C).

### The role of the SIRT1/FOXO and MEK pathways in mediating the enhanced exercise in AC5 KO mice

**Figure 7:**
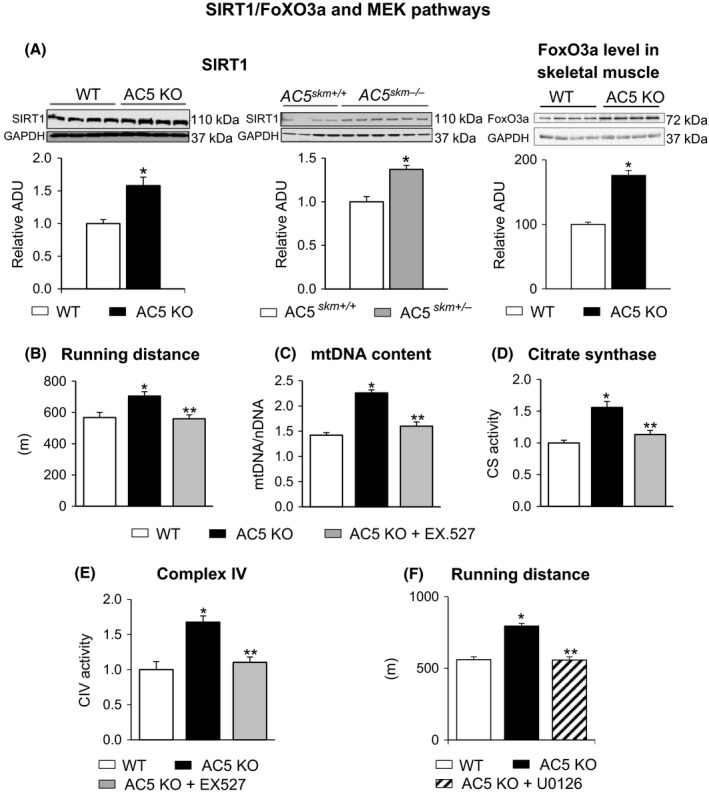
Enhanced Exercise in AC5 KO mice through SIRT1 and MEK pathways. (A) Increased SIRT1 expression in gastrocnemius muscles of AC5 KO and AC5 SKM KO compared to WT mice, and FoxO3a expression was increased in skeletal muscle in AC5 KO compared to WT mice; *n* = 4–6. The effects of SIRT1 inhibition on exercise capacity in AC5 KO mice (B–E). (B) Enhanced exercise capacity in AC5 KO mice was abolished after treatment with SIRT1 inhibitor, EX 527. Only running distance is shown, but running time, maximum speed, and work to exhaustion were all attenuated similarly to running distance by the SIRT1 inhibitor. (C–E) Increased mitochondrial biogenesis, as reflected by mitochondrial DNA content, as well as increased citrate synthase and complex IV activities in AC5 KO mice, was abolished after treatment with the SIRT1 inhibitor, EX 527. *P <* *0.01 vs. WT and **P <* *0.05 vs. AC5 KO using one‐way ANOVA. There was no significant difference between AC5 KO treated with EX 527 and WT mice. Results are expressed as the mean ± SEM. (F) The effects of MEK inhibition on exercise capacity are shown in AC5 KO mice. Enhanced exercise capacity in AC5 KO mice was abolished after treatment with the MEK inhibitor, U0126. Exercise capacity in WT remained similar after treatment with MEK inhibitor. AC, adenylyl cyclase; KO, knock out.

Gastrocnemius muscles in whole body AC5KO contained higher levels of SIRT1 and FoxO3a protein than that of the WT (Fig. 7A). To further confirm that the SIRT1 pathway was involved in the enhanced exercise capacity of AC5 KO mice, AC5 KO mice were treated with EX527, a selective SIRT1 inhibitor (Napper *et al*., 2005), which abolished the enhanced exercise capacity of AC5 KO mice (Fig. 7B). Running time, maximum speed, and work to exhaustion were attenuated similarly to running distance. This inhibitor also abolished mitochondrial biogenesis in AC5 KO mice, as reflected by reduced mitochondrial DNA content, as well as citrate synthase and complex IV activities (Fig. 7C–E). To further confirm that the MEK pathway was involved in the enhanced exercise capacity of AC5 KO mice, AC5 KO mice were treated with U0126, a selective MEK inhibitor, which also abolished the enhanced exercise capacity of AC5 KO mice (Fig. 7F).

## Discussion

The major finding of this investigation is that disruption of AC5, which actually decreases sympathetic tone, increases exercise performance. This is novel, as the most common mechanism mediating enhanced exercise is via increased sympathetic stimulation and catecholamines, resulting in increased AC activity and augmented cardiac output (Esposito *et al*., 2008). This was not the mechanism in AC5 KO mice, where AC activity is actually reduced, and there was no greater increase in cardiac output during exercise compared with WT mice, based on direct measurements of ascending aortic blood flow (stroke volume) with implanted ultrasonic flow probes and heart rate in chronically instrumented mice. Further confirming the lack of a cardiac mechanism, the cardiac‐specific AC5 KO did not exhibit enhanced exercise. Accordingly, the mechanism resided at the level of the exercising skeletal muscles, which was confirmed, when we found that exercise performance was also elevated in the skeletal muscle‐specific AC5 KO.

Exercise plays an essential role in longevity, in general, and healthful aging, in particular, as it protects not only against obesity, diabetes, and cardiovascular disease, but also reduces the risk of cancer and improves bone health and even mental diseases that impair aging (Lee & Paffenbarger, 2000; Gremeaux *et al*., 2012; Reimers *et al*., 2012). Therefore, the demonstration of improved exercise performance in the AC5 KO model is particularly germane, as this is also a model for longevity (Yan *et al*., 2007), and protects against cardiovascular stress (Okumura *et al*., 2003; Lai *et al*., 2013), diabetes, and obesity (Ho *et al*., 2015). In view of the important link between exercise and longevity, it is surprising that of 20 mouse models we reviewed, only two studied exercise and found it to be increased.

Increased mitochondrial biogenesis is known to improve exercise performance (Rockl *et al*., 2007; Yamamoto *et al*., 2011). In the present investigation, using a mitochondrial DNA content assay, skeletal muscle of AC5 KO mice was shown to contain more mitochondria. Mitochondrial function was also increased, as reflected by increased ATP content, citrate synthase activity, and complex IV activity, in AC5 KO muscle. Furthermore, levels of mitochondrial gene expression were also enhanced in skeletal muscle of AC5 KO mice. The fact that we could recapitulate many of our *in vivo* observations in skeletal muscle myoblasts after AC5 KD further indicated the cell autonomous nature of these effects. Importantly, in AC5 knockdown L6 myoblast cells, the increased real‐time O2 consumption rate supports the improved mitochondrial biogenesis and function in the skeletal muscle AC5 KO *in vivo*. Altogether, our results indicate that improved mitochondrial number and function are involved in mediating the enhanced exercise capacity of AC5 KO mice.

The SIRT1/FoxO and MEK pathways, which we showed were upregulated in AC5 KO, have also been shown to mediate mitochondrial biogenesis and oxidative stress (Kops *et al*., 2002; Brunet *et al*., 2004). The SIRT1 and MEK pathways have also been shown to mediate increased exercise performance (Dufresne *et al*., 2001; Chalkiadaki *et al*., 2014), although the role of Foxo3a is novel to the current investigation. We confirmed that these pathways were also involved in mediating the enhanced exercise performance in the AC5 KO mouse by blocking SIRT1 activity using a specific SIRT1 inhibitor, EX527, which abolished not only the enhanced exercise capacity, but also mitochondrial biogenesis, in AC5 KO mice. The involvement of the Raf/MEKERK signaling pathway was confirmed by blocking MEK with a specific inhibitor, U0126. AMPK, which like AC5 KO reduces AC, has also been shown to be linked to the SIRT1 and MEK pathways (Canto *et al*., 2009; Suchankova *et al*., 2009).

Another key finding of the current investigation was demonstrating that protection against oxidative stress, by increased MnSOD levels and activity in AC5‐deficient skeletal muscles, is also involved in the mechanism of enhanced exercise capacity in AC5 KO mice, as exercise capacity of AC5 KO mice was significantly attenuated in AC5 KO × MnSOD heterozygous KO bigenic mice. As MnSOD is a downstream target of SIRT1 (Lai *et al*., 2013), and FoxO3a is known to regulate MnSOD transcriptionally, it is likely that AC5 regulates expression levels of MnSOD in skeletal muscle also through SIRT1 and FoxO3a. It should be noted that although most studies indicate that antioxidants protect against oxidative stress and thus extend lifespan (Anisimov *et al*., 2011; Niu *et al*., 2013) and lead to enhanced exercise capacity (Ji *et al*., 1998), recently some reports indicated that antioxidants have no beneficial effects on exercise or are even harmful in high concentrations (Selman *et al*., 2013). In addition, although a number of studies have reported that exercise leads to increased longevity (Holloszy, 1998; Navarro *et al*., 2004; Barnes, 2015), one study in mice is not in agreement (Garcia‐Valles *et al*., 2013). However, the overwhelming preponderance of data in patients support the concept that exercise improves not only healthy lifespan, but also longevity, as it clearly is protective against cardiovascular disease, diabetes, and obesity, all of which are known to reduce lifespan. As the AC5 KO mice exhibit longevity and protection against diabetes, obesity, and cardiovascular disease (Yan *et al*., 2007, 2014; Lai *et al*., 2013; Ho *et al*., 2015) along with enhanced exercise performance demonstrated in the current investigation, this model replicates the human paradigm of healthful aging. Conversely, enhanced β adrenergic signaling results in diminished lifespan, not only in transgenic animal models, for example, overexpression of Gsα (Iwase *et al*., 1996, 1997), but also in human aging studies with increased β2 adrenergic receptor genotype, where longevity is diminished (Zhao *et al*., 2012).

Further support for the role of AC5 as a key gene involved in healthy aging comes from our studies in *C. elegans*, where the *acy‐3* (the worm homolog of AC5) mutant showed improved mitochondrial metabolism and antioxidative stress defense, via the induction of the expression of *sod‐3,* the worm homolog of MnSOD. The evolutionary conservation of the pathway involving decreased AC5 and increased MnSOD further testifies to the importance and essential nature of this signaling mechanism. Notably, we and others have previously shown that both enhanced mitochondrial metabolism and stress defense pathways are heavily controlled by *sir‐2.1* the worm homolog of SIRT1 (Berdichevsky *et al*., 2010; Mouchiroud *et al*., 2013). The evolutionary conservation of the AC5/SIRT1 signaling pathway further testifies to the importance and essential nature of this signaling mechanism.

One question that arose is whether these effects of enhanced exercise in AC5 KO mice are simply due to a decrease in AC, which might be evoked in a KO from any of the 9 AC isoforms, or are they due to unique signaling in AC5. To address this question, we examined exercise in 10 AC6 KO mice and 7 WT controls. The AC6 KO mice did not show increased distance (497 m) or speed (30 m min^−1^) with exercise compared to their WT (distance (510 m) or maximal speed (30 m min^−1^), and results in AC6 KO WT were not different from those in AC5 KO WT (Fig. 1). Therefore, the enhanced exercise was not simply due to a reduction in AC, but was rather unique to the AC5 KO and its signaling pathway noted above.

In summary, we discovered a novel pathway for augmenting exercise performance, which does not involve increased sympathetic tone, but actually reduced AC activity and beta adrenergic receptor signaling, by inhibiting AC5. Mechanistically, AC5 loss‐of‐function leads to enhanced exercise performance due to increased mitochondrial function and inhibition of oxidative stress through SIRT1, FoxO3a, MEK, and MnSOD. As in heart failure, exercise tolerance is markedly diminished and is actually an early diagnostic sign, which precedes the overt manifestation of the inherent cardiac defect at rest, the development of a new therapeutic modality based on the salutary effects of the AC5 KO would be significant, not only to maintain fitness, but could also to ameliorate diseases such as heart failure, diabetes, obesity, and other chronic illnesses, which limit healthful aging.

## Experimental procedures

### Animal experimental procedures

All experiments were performed in 3‐ to 6‐month‐ and 20‐month‐old systemic AC5 KO, 3‐ to 6‐month‐old skeletal muscle or cardiac‐specific AC5 KO and their corresponding WT littermates. For exercise studies, AC5 KO and WT were matched for body weight. For SIRT1 inhibition, the SIRT1 inhibitor, EX527 (Sigma‐Aldrich, St. Louis, MO, USA) was delivered to AC5 KO and wild‐type (WT) control littermates at a dose of 10 mg kg^−1^ day^−1^ with a mini‐osmotic pump (ALZET model 2001, DURECT Corp., Cupertino, CA, USA) for 7 days. For MEK inhibition, the MEK inhibitor, U0126 (Sigma), was delivered to AC5 KO and wild‐type (WT) control littermates at a dose of 10 mg kg^−1^ day^−1^ with a mini‐osmotic pump (ALZET model 2001, DURECT Corp) for 7 days. To induce oxidative stress, AC5 KO and WT mice were treated with paraquat (35 mg kg^−1^, i.p.) for 10 days. After treatment, the mice were subjected to exercise twice at intervals of 2 days. The indices of exercise capacity were measured as described above, and the extent of oxidative stress was measured by 8‐hydroxy‐deoxyguanosine (8‐OHdG) staining in the gastrocnemius muscle. Animals used in this study were maintained in accordance with the Guide for the Care and Use of Laboratory Animals (National Research Council, Eighth Edition 2011). These studies were approved by the Institutional Animal Care and Use Committee of Rutgers University—New Jersey Medical School.

### Exercise protocol and indices of exercise capacity

Mice were exercised on a treadmill (Omnitech Electronics, Columbus, OH, USA) with metabolic chambers to measure indices defining exercise capacity. All mice were subjected to a practice trial 3 days before the experiment to adapt to the treadmill testing environment. Food was withdrawn at least 3 h before the exercise. At the time of the experiment, each mouse was placed on a treadmill at a constant 10° angle enclosed by a metabolic chamber through which air flow passes at a constant speed. Then, the treadmill was started at 4 m min^−1^ and the speed incrementally increased 2 m min^−1^ every 2 min until the mice reached exhaustion. Exhaustion was defined as spending time (10 s) on the electric stimulus platform without attempting to re‐engage the treadmill belt. The maximal running speed and distance were calculated. The work to exhaustion was calculated based on the vertical running distance to exhaustion and body weight.

### Cardiac output during exercise

AC5 KO and WT mice were chronically instrumented with transonic flow probes on the ascending aorta to allow for beat‐by‐beat measurements of stroke volume, which were integrated over time, measuring cardiac output (the product of heart rate and stroke volume) at rest and during exercise.

### *Caenorhabditis elegans* experiments

*Caenorhabditis elegans* strains were cultured at 20 °C on nematode growth media agar plates seeded with *E. coli* strain. Strains used were WT Bristol N2, KN259 (huIs33[*sod‐3*::GFP + pRF4(*rol‐6(su1006)*)]), SJ4100 (zcIs13[*hsp‐6*::GFP]), SJ4005 (zcIs4[*hsp‐4*::GFP]), SJ4143 (zcIs17[*ges‐1*::GFP(mit)]), and SJ4103 (zcIs14[*myo‐3*::GFP(mit)]). Strains were provided by the *Caenorhabditis* Genetics Center (University of Minnesota). The clone used was *acy‐3* (C44F1.5) and was purchased from GeneService and sequenced. *Caenorhabditis elegans* strains, RNAi feeding experiments, GFP expression analysis, GFP imaging microscopy, and O_2_ consumption measurements using Seahorse XF24 were performed according to standardized procedures (Mouchiroud *et al*., 2013).

### Measurement of cellular respiration and metabolic rates

Generation of AC5 shRNA adenovirus (Ad‐AC5 shRNA) was described previously (Lai *et al*., 2013). Ad‐AC5 shRNA was added to L6 rat skeletal muscle cells with 80% confluence, following 72–96 h of incubation. Real‐time O2 consumption rates (OCR) in L6 myoblast cells infected with either Ad‐AC5 shRNA or Ad‐LacZ were measured with Seahorse XF24 as described (Yamamoto *et al*., 2011).

### Histological analyses

8‐OHdG staining with anti‐8‐hydroxy‐2′‐deoxyguanosine (8‐OHdG) antibody (Oxis International, Inc.) has been described previously (Yamamoto *et al*., 2003).

### Biochemical analyses

*Quantitative RT‐PCR* Total RNA was prepared from frozen heart tissues or cell cultures using Trizol reagent (Sigma). The mRNA of interest was reverse transcribed according to standard protocol. Quantitative real‐time PCR (7700 Prizm, Perkin‐Elmer/Applied Biosystems, Waltham, MA, USA) was performed with specific primers. Results were normalized to beta‐actin.

*Immunoblotting Proteins* separated by SDS‐PAGE were transferred to nitrocellulose membranes. The membranes were probed with primary antibodies to complex I, II, III, V (MitoSciences, Eugene, OR, USA), SIRT1 (Cell Signaling, Danvers, MA, USA), FoxO3a (Cell Signaling), and MnSOD (Sigma‐Aldrich, St. Louis, MO, USA ) at 4 °C overnight. The bands were visualized using chemiluminescence reagents. The linear range of detection for different proteins and band intensities were determined by densitometry. Blots were reprobed with GAPDH to equalize sample loading.

### Statistical analysis

All data are expressed as mean ± SEM. To compare two independent groups, we used Student's unpaired *t*‐test. For a comparison of three or more groups, one‐way analysis of variance (ANOVA) was used.

## Author contributions

Worm experiments performed in JA laboratory by LM and RH. Mouse experiments were performed in DV and SV laboratories by CY, LL, RP, JD, and LY. Study design was funded by DV and SV. Manuscript was written by DV, SV, LY, LM, JA, JZ, and RP. All authors have declared that no conflict of interest exists.

## Funding

This work was supported by NIH grants 5P01AG027211, 1R01HL102472, 5T32HL069752, 5P01HL069020, R01HL106511, R01HL093481, and 1R01HL119464.

## Conflict of interest

None declared.
